# Impact of Mesenchymal Stromal Cells and Their Extracellular Vesicles in a Rat Model of Kidney Rejection

**DOI:** 10.3389/fcell.2020.00010

**Published:** 2020-01-29

**Authors:** Maria Jose Ramirez-Bajo, Jordi Rovira, Marta Lazo-Rodriguez, Elisenda Banon-Maneus, Valeria Tubita, Daniel Moya-Rull, Natalia Hierro-Garcia, Pedro Ventura-Aguiar, Federico Oppenheimer, Josep M. Campistol, Fritz Diekmann

**Affiliations:** ^1^Laboratori Experimental de Nefrologia i Trasplantament (LENIT), Institut d’Investigacions Biomèdiques August Pi i Sunyer (IDIBAPS), Barcelona, Spain; ^2^Red de Investigación Renal (REDINREN), Madrid, Spain; ^3^Laboratori Experimental de Nefrologia i Trasplantament (LENIT), Fundació Clínic per la Recerca Biomèdica (FCRB), Barcelona, Spain; ^4^Departament de Nefrologia i Trasplantament Renal, Hospital Clínic de Barcelona, Barcelona, Spain

**Keywords:** bone marrow, adipose tissue, mesenchymal stromal cells, extracellular vesicles, kidney transplantation, immunomodulation, chronic kidney disease

## Abstract

**Background:**

Mesenchymal stromal cells (MSCs) from different sources possess great therapeutic potential due to their immunomodulatory properties associated with allograft tolerance. However, a crucial role in this activity resides in extracellular vesicles (EVs) and signaling molecules secreted by cells. This study aimed to evaluate the immunomodulatory properties of donor and recipient MSCs isolated from adipose tissue (AD) or bone marrow (BM) and their EVs on kidney outcome in a rat kidney transplant model.

**Methods:**

The heterotopic-kidney-transplant Fisher-to-Lewis rat model (F-L) was performed to study mixed cellular and humoral rejection. After kidney transplantation, Lewis recipients were assigned to 10 groups; two control groups; four groups received autologous MSCs (either AD- or BM- MSC) or EVs (derived from both cell types); and four groups received donor-derived MSCs or EVs. AD and BM-EVs were purified by ultracentrifugation. Autologous cell therapies were administered three times intravenously; immediately after kidney transplantation, 4 and 8 weeks, whereas donor-derived cell therapies were administered once intravenously immediately after transplantation. Survival and renal function were monitored. Twelve weeks after kidney transplantation grafts were harvested, infiltrating lymphocytes were analyzed by flow cytometry and histological lesions were characterized.

**Results:**

Autologous AD- and BM-MSCs, but not their EVs, prolonged graft and recipient survival in a rat model of kidney rejection. Autologous AD- and BM-MSCs significantly improved renal function during the first 4 weeks after transplantation. The amelioration of graft function could be associated with an improvement in tubular damage, as well as in T, and NK cell infiltration. On the other side, the application of donor-derived AD-MSC was harmful, and all rats died before the end of the protocol. AD-EVs did not accelerate the rejection. Contrary to autologous MSCs results, the single dose of donor-derived BM-MSCs is not enough to ameliorate kidney graft damage.

**Conclusion:**

EVs treatments did not exert any benefit in our experimental settings. In the autologous setting, BM-MSCs prompted as a potentially promising therapy to improve kidney graft outcomes in rats with chronic mixed rejection. In the donor-derived setting, AD-MSC accelerated progression to end-stage kidney disease. Further experiments are required to adjust timing and dose for better long-term outcomes.

## Introduction

Patient survival after kidney transplantation has improved over the past decade due to the optimization of immunosuppressive strategies. Unfortunately, these treatments can cause severe side effects, including immunologic side effects, post-transplant malignancy, opportunistic infections and non-immunologic side effects such as nephrotoxicity and neurotoxicity (Yu et al., 2017). Thus, it is necessary to study new strategies to minimize immunosuppression or novel interventions such as cell-based therapies to modulate the immune response promoting a state of tolerance in organ transplantation and the graft survival (Sayegh and Remuzzi, 2007; Crop et al., 2009; Casiraghi et al., 2016; Rovira et al., 2017).

The application of cell therapies as immunomodulatory strategies in a clinical setting is promising. Concretely, mesenchymal stromal cells (MSCs) have beneficial proprieties against inflammation (Wise and Ricardo, 2012), apoptosis (Sung et al., 2013; Liu et al., 2015), fibrosis (Caldas et al., 2008; Choi et al., 2009) and also immunomodulation activities (Bartholomew et al., 2002; Ben-Ami et al., 2014; Insausti et al., 2014) in various *in vivo* models of ischemia/reperfusion (Togel et al., 2005; Chen et al., 2011), and renal allograft rejection (Reinders et al., 2010; Hara et al., 2011; Franquesa et al., 2012; Cao et al., 2013), without adverse events reported. Donor-derived MSCs therapy could be especially interesting due to low immunogenicity when compared with other donor-derived cell types from healthy donors (Lohan et al., 2017). However, autologous MSC therapy could be a safer choice to avoid immune responses. In addition, one of the challenges is to find the most appropriate stem cell type, since proliferation capacity and secretion of secreted paracrine factors depend on the cell type. Bone marrow-MSCs (BM-MSCs) are the most widely studied; however, they are not always the most interesting option. The immunomodulatory properties of MSCs from different adult human tissues; adipose-derived (AD), umbilical cord blood (CB), and cord Wharton’s jelly (WJ), showed an equivalent potential to suppress T-cell proliferation (Ammar et al., 2015; Pleumeekers et al., 2018) and a different capacity for differentiation (Liu et al., 2007), secretion of different paracrine factors, as VEGF-D, IGF-1, IL-8, and IL-6, that contributes to different levels of angiogenic capacity (Hsiao et al., 2012).

Previous studies showed that in addition to cell contact, the action of MSCs is due to paracrine signaling induced by the secretion of cytokines, growth factors and extracellular vesicles (EVs). However, their mechanisms of action remain unclear. EVs are tiny membrane-enclosed droplets released by cells through membrane budding and exocytosis and are composed of several cytoplasmatic components. They represent a cell-cell paracrine/endocrine communication mechanism allowing the transfer of inflammatory cytokines, growth factors and microRNAs which can regulate the proliferation, maturation, and migration of different types of immune cells (Seo et al., 2019). MSC-EVs could reproduce the immunomodulatory functions of MSCs targeting T cells (Blazquez et al., 2014; Del Fattore et al., 2015), B cells (Budoni et al., 2013) and NK cells (Di Trapani et al., 2016) and reduce the production of pro-inflammatory cytokines (Ma et al., 2019). Besides, the MSC-EVs compared with the MSCs are a safe cell-free alternative with advantages regarding immunogenicity and tumorigenicity.

In this study, we show for the first time a full comparison of the therapeutic effect of AD- and BM-MSC and their EVs within autologous or donor-derived settings in a rat model of chronic kidney allograft rejection.

## Materials and Methods

### Animals

Male Lewis rats received male either Lewis or Fischer-344 (Fisher) grafts for syngeneic and donor-derived kidney transplants, respectively. Fisher and Lewis strains differ partially at major histocompatibility complexes and various non-MHC loci, conferring a weak histocompatible combination. The animals were kept at a constant temperature, humidity, and at a 12-h light/dark cycle with free access to water and rat chow. The study was approved by and conducted according to the guidelines of the local animal ethics committee (Comitè Ètic d’Experimentació Animal, CEEA, Decret 214/97, Catalonia, Spain).

### Isolation of Mesenchymal Stromal Cells From Adipose Tissue (AD-MSC) and Bone Marrow (BM-MSC)

Perirenal AD-MSCs were obtained from Lewis or Fisher rats (200*g*). Adipose tissue was cleaned with PBS, minced, and digested with 0.10% collagenase type IV (Thermo Fisher Scientific, Waltham, MA) in modified Eagle’s medium (α-MEM) for 2 h at 37°C. The cell suspension was centrifuged at 400*g* for 20 min at room temperature. Cells were seeded and expanded in α-MEM supplemented with 10% fetal bovine serum (FBS).

Bone marrow-MSCs were isolated from femurs of Lewis or Fisher rats (200g). The bone shaft was extracted inserting a 22G needle and flushed out with α-MEM supplemented with 10% FBS and 2 mM EDTA. The cell suspension was centrifuged at 400*g* for 20 min at room temperature. Cells were seeded and expanded in α-MEM supplemented with 10% FBS.

After 48 h, non-adherent cells from AD- and BM-MSCs cultures were removed and fresh medium was replaced. Cells were cultured continuously for 1 to 3 weeks and then trypsinized. Subsequently, MSCs were frozen and cryopreserved in α-MEM medium supplemented with 10% FBS, and 10% dimethyl sulfoxide (DMSO). Three to five days before MSC administration, cells were thawed, seeded and cultured to ensure their viability. The day of administration MSC cells were trypsinized and prepared in physiological saline.

#### Characterization of AD- and BM-MSCs by Flow Cytometry

Cells had a typical spindle-shaped appearance and phenotype was confirmed by expression of MSC markers (CD44H, CD29, and CD90) and the absence of markers of the hematopoietic and endothelial lineage (CD45 and CD31, respectively) by flow cytometry (Supplementary Figure S1A). The cell suspension was stained with the antibodies indicated in Supplementary Table S1. Flow cytometry analysis was performed on a FACS Canto II (BD Biosciences, Heidelberg, Germany) and data were analyzed using FlowJo software (Tree Star, Ashland, OR, United States).

#### Osteogeneic and Adipogenic Differentiation of AD- and BM-MSCs

The potential of AD- and BM-MSCs to differentiate into adipogenic and osteogenic lineages were examined. To induce adipogenic differentiation, cells were treated with an adipogenic differentiation medium (Lonza, Basel, Switzerland) for 3 weeks. Adipogenesis was assessed by Oil Red O staining (Supplementary Figure S1B). For osteogenic differentiation, cells were treated with osteogenic differentiation medium (Lonza, Basel, Switzerland) for 3 weeks. Osteogenesis was assessed by Alizarin Red S staining (Supplementary Figure S1C). Medium changes were performed twice weekly for the two assays.

### Isolation of Extracellular Vesicles Derived From MSCs

EVs were isolated from supernatants of AD- or BM-MSCs cultured during 16 h in RPMI1640 deprived of FBS at 37°C. The supernatant was centrifuged at 3000*g* for 20 min to remove cell debris and apoptotic bodies followed by microfiltration with 0.22μm pore filter membranes. Cell-free supernatants were centrifuged at 100,000*g* for 1 h at 4°C. EV pellets were resuspended in medium RPMI1640 supplemented with 10% DMSO and frozen at –80°C for later use (Herrera et al., 2010). According with “Minimal Information for Studies of Extracellular Vesicles 2018 (MISEV2018)”, we have prepared the checklist (Supplementary Material Information).

#### Characterization of MSC-EV by Electronic Microscopy

A Holey Carbon support film on a 400-mesh copper grid was used. After glow discharge, the sample was deposited onto the grid, which was mounted on a plunger (Leica EM GP) and blotted with Whatman No. 1 filter paper. The suspension was vitrified by rapid immersion in liquid ethane (–179°C). The grid was mounted on a Gatan 626 cryo-transfer system and inserted into the microscope. Images were obtained using a Jeol JEM 2011 cryo-electron microscope operated at 200kV, recorded on a Gatan Ultrascan US1000 CCD camera and analyzed with a Digital Micrograph 1.8 (*n* = 3 per group) (Supplementary Figures S2A,B).

#### Characterization of MSC-EV by NanoSight

Size distribution and concentration of EVs were measured using the NanoSight LM10 instrument (Malvern, United Kingdom), equipped with a 638 nm laser and CCD camera (model F-033). Data were analyzed with the Nanosight NTA Software version 3.1 (build 3.1.46), with detection threshold set to 5, and blur, Min track Length, and Max Jump Distance set to auto. Samples were evaluated using different dilutions in sterile-filtered PBS 1X. Readings were taken in single capture or triplicates during 60 s at 30 frames per second (fps), camera level at 16 and manual monitoring of temperature. Supplementary Figures S2C,D shows representative results obtained by NanoSight from EVs produced by AD- and BM-MSCs.

#### Characterization of MSC-EV by Flow Cytometry

The size of EVs was calculated with Megamix-Plus SSC beads (BioCytex, Marseille, France) that contain a mix of green fluorescent bead populations with sizes of 160, 200, 240, and 500 nm. The analysis was performed using a log scale for forward scatter and side scatter parameters, and a threshold SSC-H of 1000. MSC-derived EVs were characterized by flow cytometry according MSCs markers (CD44^+^, CD29^+^, CD90^+^, CD45^–^, CD31^–^) and EVs markers (CD9^+^ and CD81^+^) (Supplementary Table S1 and Supplementary Figure S3).

### Fisher-to-Lewis Renal Transplant Model

For renal transplantation, Lewis rats received either male Lewis or Fisher grafts for syngeneic (L-L) and donor-derived renal transplants (F-L), as previously described (Rovira et al., 2018).

### Experimental Design and Follow-Up

Recipients Lewis rats of allogenic kidney grafts were distributed in nine groups after transplantation (F-L, *n* = 126). (1) Control group (F-L + Ø, *n* = 42); (2–5) autologous cell therapies groups: F-L + AD-MSCs (*n* = 11), F-L + BM-MSCs (*n* = 11), F-L + AD-EVs (*n* = 19), and F-L + BM-EVs (*n* = 14); (6–9) donor-derived cell therapies groups: F-L + AD-MSCs (*n* = 5), BM-MSCs (*n* = 4), AD-EVs (*n* = 10), and BM-EVs (*n* = 10). Eleven Lewis rats received a syngeneic kidney graft (L-L).

**FIGURE 1:**
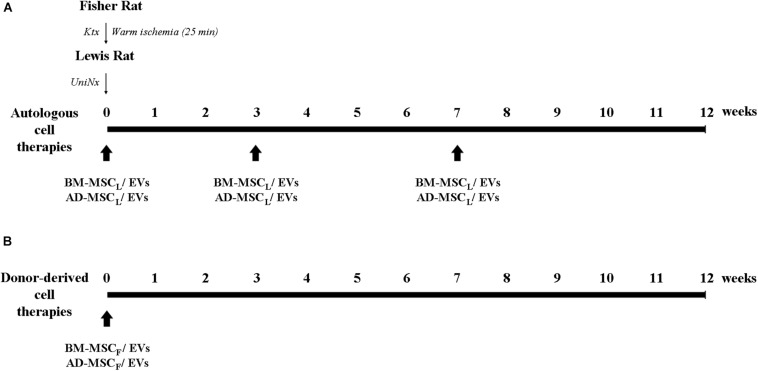
Experimental design of the protocol of AD- and BM-MSCs and their EVs administration regimens from recipient and donor in the Fisher-Lewis renal transplant model. **(A)** Cell therapies from recipients were administered intravenously in the moment of the transplant, and 4 and 8 weeks post-transplant. **(B)** Cell therapies from the donor were administrated once after kidney transplantation. KTx, kidney transplantation; UniNx, right uninephrectomy; AD, adipose tissue-derived; BM, bone marrow; MSC, mesenchymal stromal cells; EVs, extracellular vesicles.

For autologous cell therapies, 1 × 10^6^ MSCs or 1.4 × 10^9^ EVs from Lewis rats were resuspended in 400 μl of physiological saline and injected through the tail vein at the moment of the kidney transplantation, and 4 and 8 weeks after transplantation. For donor-derived cell therapies, Lewis rats received one intravenous dose of AD-, BM-MSCs or their EVs immediately after transplantation (Figure 1). One week after transplantation and monthly until the end of the study, at 12 weeks, rats were weighed and placed in metabolic cages for 24 h urine and tail-vein-blood collection. From blood samples, BUN and blood creatinine were determined, and urine creatinine and proteinuria from 24h urine.

At the end of the study, the animals were sacrificed and kidney graft and spleen were harvested. Then the kidney graft was divided: one piece was fixed in formalin and another piece was used for infiltrating immune cell detection by flow cytometry.

### Histology and Immunohistochemistry Analysis

Paraffin-embedded renal sections (3 μm-thick) were stained with hematoxylin-eosin (H/E), periodic acid Schiff (PAS) and Sirius Red. These stains were evaluated by a renal pathologist (Eduardo Vazquez-Martul) and the degree of tubular atrophy (TA), fibrosis (F), tubulitis, peritubular capillaritis (PTC) and glomerular pathology was quantified. TA and F were scored from grade 0 to 3 (0, none [<10%]; 1, mild [10 to 20%]; 2, moderate [20 to 50%]; 3, severe [>50%]). Tubulitis was scored from grade 0 to 3 according to Banff criteria (1, <4 lymphocytes per tubular section and 2, >4 lymphocytes). PTC was scored from grade 0 to 3 according Gibson-Banff classification (1, <3 monocytes/ptc lumen; 2, 3-5 monocytes/ptc lumen; 3, >5 monocytes/ptc lumen).

Sections (3 μm-thick) mounted on xylene glass slides were used for immunohistochemistry. After antigen retrieval had been carried out, endogenous peroxidase blocking for 10 min in 3% hydrogen peroxide (Merck, Darmstadt, Germany) was performed before primary antibody incubation. The primary antibody, rat anti-C4d (Hycult Biotech, Plymouth Meeting, PA, United States) was incubated overnight at 4°C. Envision system-specific anti-rabbit secondary antibody labeled with horseradish peroxidase polymer (Dako, Glostrup, Denmark) was applied for 1 h. All sections were counterstained with Mayer’s hematoxylin. The immunohistochemical procedure was performed at the same time to avoid possible day-to-day variations in staining performance. All images were acquired using an Olympus BX51 clinical microscope and DP70 digital camera and software (Olympus, Tokyo, Japan).

### Flow Cytometric Characterization of Immune Cells

Spleens were mashed and passed through a 70 μm nylon cell strainer (BD Falcon) and single-cell suspensions were obtained. Kidney grafts were digested with collagenase type IV (Thermo Fisher Scientific, Waltham, MA, United States) and mechanically dissociated by GentleMacs (Miltenyi Biotec GmbH, Germany) to obtain a single-cell suspension. Cell surface markers were stained with antibodies indicated in Supplementary Table S2, according to the instructions of the manufacturer. Cells were stained intracellularly with Foxp3 specific mAbs using the intracellular Foxp3-staining kit (eBiosciences San Diego, CA, United States). In all the samples, Aqua Live/Dead fixable dead cell kit (Thermo Fisher Scientific, Waltham, MA, United States) was used unambiguously to remove dead cells. Flow cytometry analysis was performed on a FACS Canto II (BD Biosciences, Heidelberg, Germany). Data were analyzed using FlowJo software (Tree Star, Ashland, OR, United States). Overview of the gating strategy for T, NK, and B cells has been shown in Supplementary Figure S4.

### Statistical Analysis

Statistical analysis was performed using GraphPad Prism 5 statistical software (GraphPad Software Inc.). Univariate analysis using the log–rank test (Kaplan–Meier curves) was conducted to assess rat survival (time from kidney transplantation to death). Values are given as mean ±standard deviation. The Kruskal-Wallis or Mann-Whitney *U* tests were used where applicable.

## Results

The main results are summarized in the Table 1.

**TABLE 1:** Summary of effects of donor-derived and autologous AD- and BM-MSCs and their EVs administration regimens in the Fisher-Lewis renal transplant model at the end of the study (12 weeks).

**Treatment**	**Cell therapy**	**Renal function**	**Survival**		**Histology**			**Lymphocyte infiltration**
					
				**Tubular atrophy**	**Tubulitis**	**Capillaritis**	**Fibrosis**	
Autologous	BM-MSC_L_	=	↑	=	↓	↓	↓	**↓** T cell and NK cells
	BM-EV_L_	=	=	=	↓	=	=	**=**
	AD-MSC_L_	=	↑	=	↓	↓	↓	**↓** T cell and NK cells
	AD-EV_L_	=	=	↓	↓	↓	↓	**=**
Donor-derived	BM-MSC_F_	=	↑	↓	↓	↓	↓	**=**
	BM-EV_F_	=	=	=	↓	=	=	**=**
	AD-MSC_F_	↓	↓	Ø	Ø	Ø	Ø	Ø
	AD-EV_F_	=	=	↓	↓	↓	↓	**↓** T cell and NK cells

*=, no change; ↑, increase; ↓, decrease; Ø, no rat survives.*

### Autologous Cell Therapies

#### Renal Function

**FIGURE 2:**
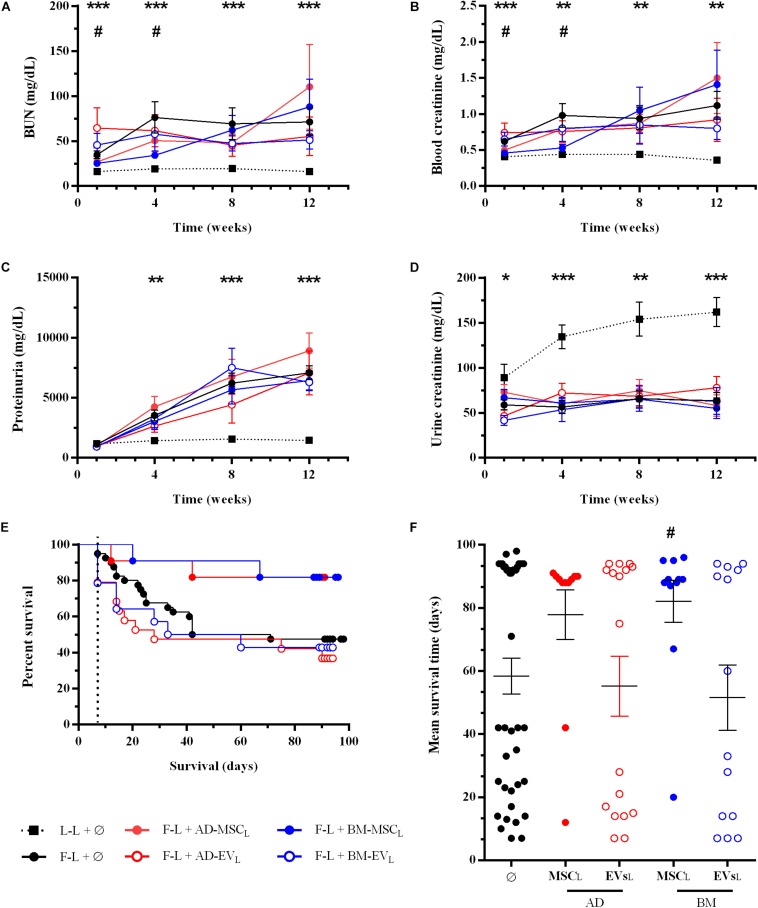
Effect of autologous AD- or BM-MSCs and their EVs treatments on renal function and rat survival after kidney transplantation. **(A)** Blood Urea Nitrogen. **(B)** Blood creatinine levels. **(C)** Proteinuria. **(D)** Urine creatinine levels. **(E)** Survival curve was generated using the Kaplan-Meier method and compared using the long-rank (Mantel-Cox) test. **(F)** Mean survival time (days). *Significantly different when compared L-L vs F-L + Ø group (*P < 0.05; **P < 0.01; and ***P < 0.001). ^#^F-L + Ø group vs BM-MSCF (^#^*P* < 0.05).

Renal function was evaluated monthly after renal transplantation. Donor-derived control group (F-L + Ø) showed progression of renal failure characterized by an increase of BUN, serum creatinine and proteinuria (PU) levels and a reduction of urine creatinine, compare to syngenic group (L-L + Ø) (Figures 2A–D). The administration of AD- or BM-MSC_L_ from Lewis rats significantly improved renal function, both BUN and blood creatinine, during the first 4 weeks after transplantation. Subsequently, these improvements weakened progressively until the end of the study. AD-MSC_L_ and their EVs showed worse results compared to BM-MSC_L_ and their EVs. None of the treatments blocked the progression of PU.

#### Survival

At the end of this mixed cellular and humoral rejection model, F-L + Ø group, 100% of Lewis recipients rejected Fisher kidney grafts and nearly 50% progressed to end-stage renal disease and died. The Kaplan-Meier plot showed that autologous AD- and BM-MSC_L_ increased rat survival from 47.5% (control group F-L) to 82% in both therapies without reaching statistical significance (*P* = 0.059 and *P* = 0.054, respectively) (Figure 2E). Nevertheless, BM-MSC_L_ treatment improved significantly mean survival time compared to the F-L + Ø group (Figure 2F). EVs therapies did not improve the survival rate nor survival time.

#### Histology and Immunohistochemistry

**FIGURE 3:**
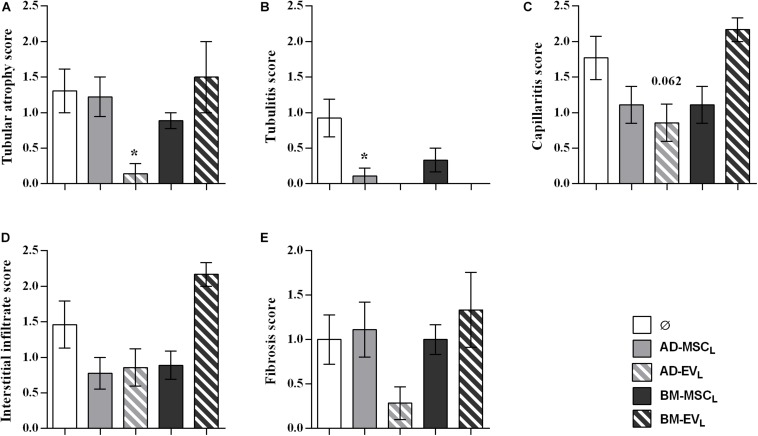
Histological evaluation of lesions observed in renal allograft during the rejection process after autologous AD-, BM-MSCs and their EVs treatments. **(A)** Tubular atrophy, **(B)** fibrosis, **(C)** tubulitis, **(D)** capillaritis, and **(E)** interstitial infiltrate in rats without treatment (Ø) and with the administration of AD-, BM-MSC, and their EVs. Significantly different when compared to F-L + Ø group (*P < 0.05).

At the end of the study, histological examination of kidneys from the control group (F-L + Ø) revealed a mixed cellular and humoral rejection characterized by moderate-to-severe tubular atrophy, tubulitis, peritubular capillaritis and fibrosis (Figure 3). EVs derived from AD-MSC_L_ reduced tubular atrophy in kidney grafts (Figure 3A). All therapies reduced tubulitis injury (Figure 3B), AD-MSC_L_ treatment reached statistical differences, and none of the kidney grafts into recipients treated with EVs either AD- and BM-MSC_L_ presented tubulitis.

Donor-derived renal transplantation (F-L) is characterized by C4d deposition in the peritubular capillaries (2.00 ± 1.00). AD- and BM-MSC_L_ therapies significantly reduced C4d deposition (0.17 ± 0.17 and 0.11 ± 0.11, respectively, and *P* < 0.0001 in both cases) (Supplementary Figure S5).

#### Lymphocyte Infiltration Into Kidney Graft and Spleen

**FIGURE 4:**
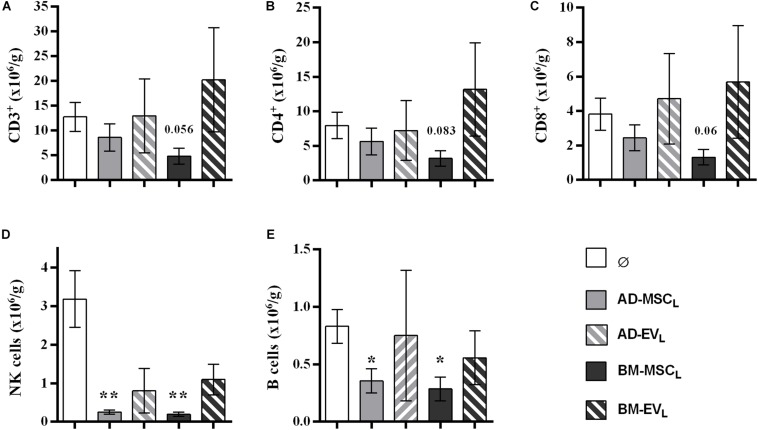
Effect of autologous AD- and BM-MSCs and their EVs treatments on infiltrating immune cell in kidney graft. **(A)** T cells, CD3^+^. **(B)** T_helpers_ + T_reg_ cells, CD3^+^CD4^+^. **(C)** T_cytotoxic_ cells, CD3^+^CD8^+^. **(D)** NK cells, CD3^–^CD314^+^CD161^+^. **(E)** B cells, CD3^–^CD161^–^B220^+^. *Significantly different when compared to F-L + Ø group (*P < 0.05; **P < 0.01).

The analysis of infiltrating lymphocytes in the kidney graft showed that the recipient AD- and BM-MSC reduced partially T-cell filtration, whereas B, NK cells were significantly reduced (Figure 4). Both EVs therapies, AD-EV_L_ and BM-EV_L_, did not modify kidney graft lymphocyte infiltration. None of the recipient cell therapies modified the spleen lymphocyte subtypes counts (Supplementary Figure S6).

### Donor-Derived Cell Therapies

#### Renal Function

**FIGURE 5:**
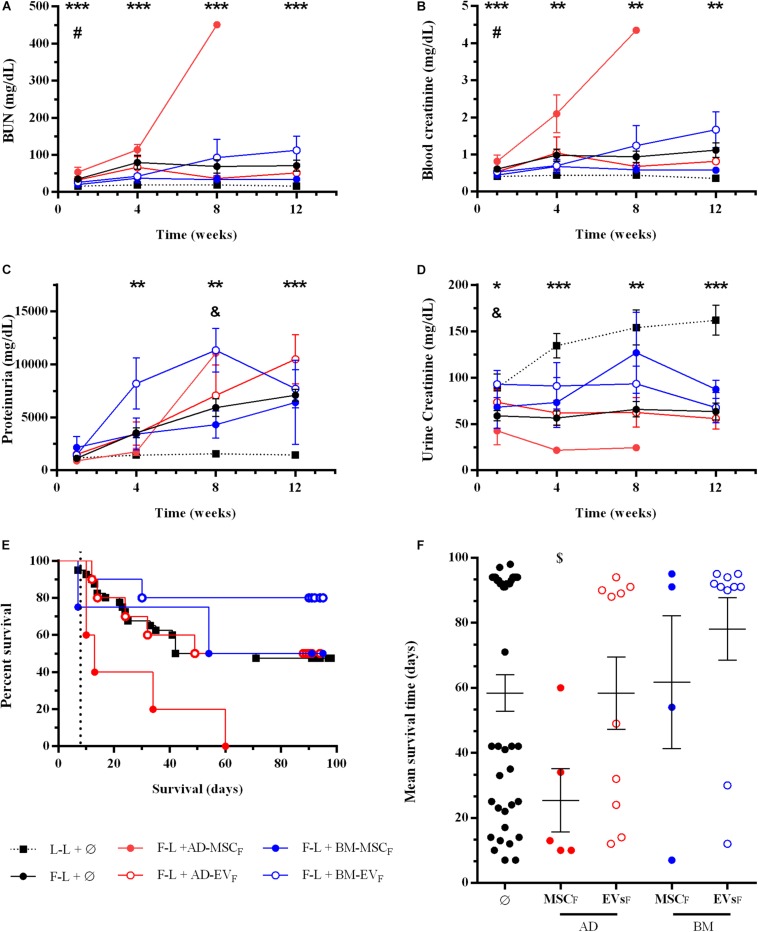
Effect of donor-derived AD- or BM-MSCs and their EVs treatments on renal function and rat survival after kidney transplantation. **(A)** Blood Urea Nitrogen. **(B)** Blood creatinine levels. **(C)** Proteinuria. **(D)** Urine creatinine levels. **(E)** Survival curve was generated using the Kaplan-Meier method and compared using the long-rank (Mantel-Cox) test. **(F)** Mean survival time (days). *Significantly different when compared L-L vs F-L + Ø group (*P < 0.05; **P < 0.01, and ***P < 0.001). ^#^F-L + Ø group vs BM-MSC_F_ (^#^*P* < 0.05). ^&^F-L + Ø group vs BM-EVF (^&^*P* < 0.05). ^[*dollar*]^F-L + Ø group vs AD-MSCF (^[*dollar*]^*P* < 0.05).

The application of donor AD-MSC_F_ impaired renal function (both BUN and blood creatinine), whereas BM-MSC_F_ improved renal function at short-term analysis (until week four after transplantation) (Figures 5A–D). The application of EVs derived from AD-MSC_F_ or BM-MSC_F_ did not modify renal function compared to the control group.

At 12 weeks after transplantation, rats treated with donor BM-MSC_F_ had slightly better renal function than the control group. The administration of EVs, independently from the tissue-derived, did not improve renal function.

#### Survival

As mentioned previously, the control group survival was 47.5% (Figures 5E,F). The application of donor-derived AD-MSC_F_ was harmful, and all rats from this group died before the end of protocol follow-up at 12 weeks (*P* = 0.0074). Donor-derived BM-MSC_F_ and AD-EV_F_ therapies did not improve the survival rate, being 50% in both groups. Eight out of ten rats treated with EVs from donor-derived BM-MSC_F_ survived until the end of the protocol, however, neither Kaplan-Meier nor mean survival time analysis confirmed the statistical differences compare to the control group.

#### Histology

**FIGURE 6:**
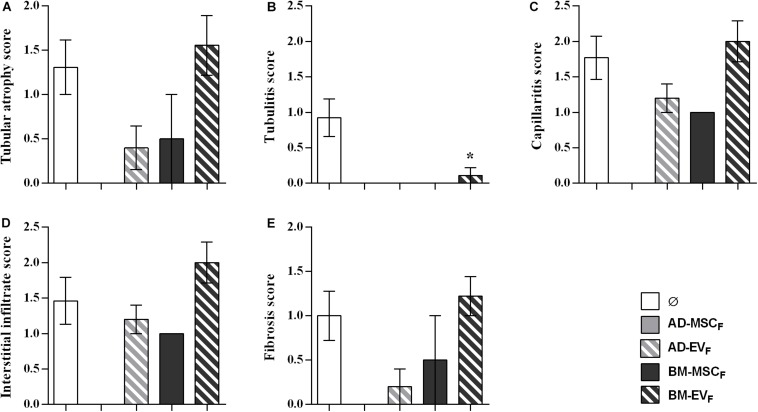
Histological evaluation of lesions observed in renal allograft during the rejection process after donor-derived BM-MSCs, their EVs and AD-EVs treatments. **(A)** Tubular atrophy, **(B)** fibrosis, **(C)** tubulitis, **(D)** capillaritis, and **(E)** interstitial infiltrate in rats without treatment (Ø) and with treatment. Significantly different when compared to F-L + Ø group (*P < 0.05).

Kidney grafts from surviving rats of AD-EV_F_ and BM-MSC_F_ groups showed a partial reduction of tubular atrophy, peritubular capillaritis, interstitial infiltrate, and fibrosis without reaching statistical differences. None of the kidney grafts from surviving rats of AD-EV_F_ and BM-MSC_F_ groups showed tubulitis, whereas kidney grafts from the BM-EV_F_ group had less tubulitis than the control group (Figure 6).

#### Lymphocyte Infiltration Into Kidney Graft and Spleen

**FIGURE 7:**
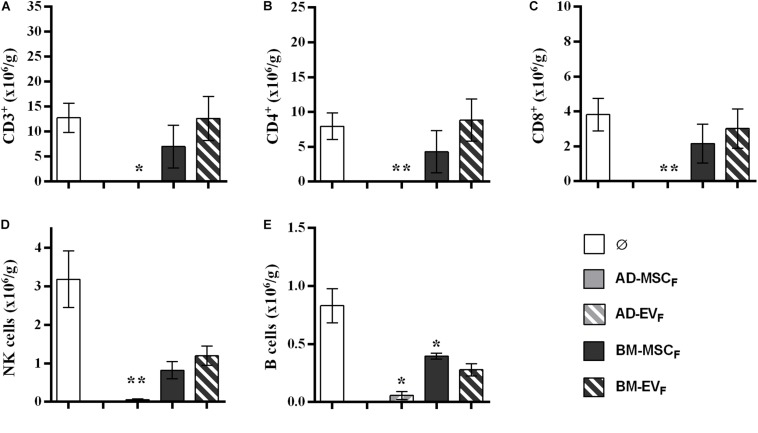
Effect of donor-derived AD- and BM-MSCs and their EVs treatments on infiltrating immune cell in kidney graft. **(A)** T cells, CD3^+^. **(B)** T_helpers_ + T_reg_ cells, CD3^+^CD4^+^. **(C)** T_cytotoxic_ cells, CD3^+^CD8^+^. **(D)**, NK cells, CD3^–^CD314^+^CD161^+^. **(E)** B cells, CD3^–^CD161^–^B220^+^. ^∗^Significantly different when compared to F-L + Ø group (*P < 0.05; **P < 0.01).

The analysis of infiltrating lymphocytes in survival kidney grafts at the end of protocol showed a reduction of T, B, and NK cell infiltration in rats treated with AD-EV_F_. None of the other cell therapies reduced T cell infiltration (Figure 7). The spleen lymphocytes’ amount and subtypes remained stable between groups (Supplementary Figure S7).

## Discussion

The Fisher-to-Lewis renal transplantation is a model of mixed cellular and humoral rejection, where kidney allografts develop clinical and histopathological features of immune-mediated chronic allograft dysfunction (White et al., 1969; Rovira et al., 2018). A large number of studies had been focusing on the application of MSCs from different sources in kidney transplants, and few studies have compared allogeneic *versus* autologous MSC therapy to determine its beneficial effects on renal rejection outcome. Moreover, stem cell-derived EVs are described as a new therapeutic option for renal injury, but their application in pre-clinical models is only related to avoiding renal damage during the organ reperfusion prior to transplant (Gregorini et al., 2017; Rigo et al., 2018). Here, we have evaluated the immunomodulatory properties of MSCs isolated from adipose tissue (AD) or bone marrow (BM) and their EVs within autologous and donor-derived settings, on kidney outcome in a rat model of kidney rejection without immunosuppression associated.

MSCs therapy is limited by their poor engraftment and rapid disappearance on most of studied models. For this reason, it is rationale suppose that multiple administrations are able to increase the efficiency of the treatment and it has to be the most recommended therapy (Tang et al., 2018). Our results showed that multiple autologous AD- and BM-MSC_L_ doses could improve the survival of animals undergoing cell therapy. Specifically, BUN and serum creatinine levels were reduced during the first 4 weeks after transplantation, however, it did not avoid kidney failure in the long-term. Rats treated with BM-MSC_L_ presented better outcomes than AD-MSC_L_. In addition, histological analysis revealed an amelioration of the tubular injury and C4d deposition related to antibody-mediated rejection that requires complement activation. Tu et al. (2010) described that MSCs are able to inhibit complement pathway activation due to the secretion of Factor H, which is a natural regulator of the complement system. This histological improvement could be related to the partial and significant reduction of T- and B-cell filtration and NK cells observed in kidney graft. Corroborating, previous *in vitro* experiments that showed a suppression by MSC of NK cell proliferation, cytolytic activity and cytokine production (Spaggiari et al., 2006; Lupatov et al., 2017). Furthermore, it has been described that the administration of MSCs *in vitro* and *in vivo* models of acute rejection in cardiac transplant induces an alteration in dendritic cell (DC) differentiation, maturation, and cytokines secretion (Nauta et al., 2006; Ge et al., 2009). In addition, B cell proliferation and differentiation to IgM and IgG producing-cells are inhibited (Corcione et al., 2006; Vogelbacher et al., 2010). Finally, MSCs mediated T cell inhibition enhancing a reduction of IFNγ and an increment of VEGF, and soluble factors (TGFβ and HGF) (Di Nicola et al., 2002). Therefore, it is evident that MSCs are receptive to signals from the environment and they have the potential to direct reprogramming of immune system cells promoting the host defense and avoiding an inflammatory process. However, in our *in vivo* model, donor-derived AD- and BM-EV_F_ did not improve the renal function, survival rate or immunomodulation, and we only observed a reduction in tubular atrophy in the kidney grafts and tubulitis. Therefore, it could be crucial in future experiments to optimize timing, doses, and frequency of cell therapy and derivatives for understanding the mechanisms and inducing the immunomodulation.

In donor-derived MSCs setting, we have considered a single administration because these cells are not intrinsically immunoprivileged since these cells could induce rejection, which is followed by an immune memory and sensitization. A couple of clinical trials, it has demonstrated that 19% and 34% of patients treated with allogeneic MSC developed HLA class I donor-specific antibodies (DSA) (Panes et al., 2016; Alvaro-Gracia et al., 2017). Our results have shown that donor-derived AD-MSC_F_ treatment is a harmful therapeutic option in the F-L kidney transplant model. AD-MSC_F_ impaired renal function (both BUN and blood creatinine) leading to graft dysfunction and finally graft loss before the end of the study. The application of a single injection from allogenic AD-MSC_F_ was able to trigger an immune response enough to induce 100% mortality to the animals. These results corroborated previous studies with murine MSCs which demonstrate that MSC not are immunoprivileged *in vivo*, at least not when there is a complete MHC class I and II mismatch (Eliopoulos et al., 2005). In spite of a single intra-articular injection of autologous MSCs did not result in an adverse clinical reaction (Carrade et al., 2011), repeated intra-articular injections of donor-derived MSCs resulted in an adverse clinical response, suggesting there is an immune recognition of donor-derived MSCs after a second exposure (Joswig et al., 2017). In our study, the recipients were submitted to a double exposure at the same time: MSCs and kidney graft, being the last one a continued and prolonged exposure. Moreover, in the case of donor-derived BM-MSC_F_, this therapy did not provide any survival benefit in our model but improved renal function at short-term analysis (until week four after transplantation), and at 12 weeks after transplantation. This slight amelioration of renal function was associated with a histological improvement, which was not sufficient to counteract the reject evolution. To try to do an explanation about this differences in immunoreactivity that we observed in this setting, it is that although they have similar surface molecular markers, and immunomodulatory capacities (Yoo et al., 2009; Pendleton et al., 2013; Hao et al., 2017), their differentiation capacity (Liu et al., 2007) as well as, the secretion of paracrine factors is different (Togel et al., 2005; Hsiao et al., 2012). It is important to highlight that most of these studies about their characterization are *in vitro* models with a relative translatability to a complex *in vivo* models. Further *in vivo* studies have to be performed to enlighten the deleterious effect of donor-derived AD-MSC.

The application of donor-derived EVs, independently from the tissue-derived, did not improve renal function. Surprising, BM-EV_F_ showed a slight improvement in survival but AD-EV_F_ was the only therapy that induced a reduction of T and NK cell infiltration into the kidney. There are controversies in the bibliography about the immunomodulatory potential of MSC-EVs. While in pre-clinical studies it is described that MSC-EVs treatment halted DC maturation resulting in decreased secretion of pro-inflammatory cytokines IFNγ, TNFα, IL1β, IL6, and IL-12p70 and increased the production of anti-inflammatory cytokine as TGFβ and IL10 (Reis et al., 2018; Ma et al., 2019). There are differences in the results obtained about B cell immunomodulation (Koppler et al., 2006; Budoni et al., 2013; Carreras-Planella et al., 2019), and this immunological capacity seems to be minor when is compared with parental cells (Conforti et al., 2014).

Our study has limitations. As EVs were isolated by ultracentrifugation, and as a consequence, the pellet obtained contains a heterogeneous population of EVs, including microvesicles and exosomes, and bioactive proteins as contaminants, that could interfere in our results. Another limitation that has to be considered is the EV’s dose administered. In the literature, the range of doses administrated, either single or multiple injections, was 30 to 100μg of protein from EVs or 5.3 × 10^7^ to 1 × 10^10^ of EVs (Grange et al., 2017, 2019). In our setting, we would like to treat the animals with an intermediate dose. For that reason, we used 1 × 10^9^ EVs that is equivalent to 60μg of protein from EVs. Moreover, in donor-derived MSCs setting, after 4 weeks of transplantation, we have observed that 25 and 60% of rats died, in BM-MSC_F_ and AD-MSC_F_, respectively. The increased mortality led to decide not to include more rats per group, according to guidelines of the local animal ethics committee.

Despite of these limitations, we believe that the improvement of MSCs therapies *versus* EVs that we observed could be due to contact cell, moreover at the constant secretion of paracrine signals as EVs and growth factors. In our opinion, for applying an EV’s therapy in the future could be necessary more *in vivo* studies to perform timing and dose of administration. In addition, the choice of autologous or donor-derived origin is complex because autologous are more compatible and can escape from rejection contrary to the donor-derived origin. However, the quality of autologous cells could differ between the patients (Bolton and Bradley, 2015; Mohamed-Ahmed et al., 2018; Chung, 2019) in contrast with donor-derived cells, and that point is very relevant in the renal improvement. In our hands, donor-derived MSC therapies showed worse results than autologous MSC therapies. Concretely, donor-derived AD-MSC accelerates the rejection process whereas autologous BM-MSC was the most promising therapy. Moreover, autologous and allogenic EVs derived from AD- or BM-MSCs did not improve renal function and graft survival. The reduction of cellular infiltrate showed could be associated with the described induction of tolerance associated with Treg dependent mechanism related to indoleamine 2, 3-dioxygenase (IDO) production induced by MSCs (Ge et al., 2010; Casiraghi et al., 2012). In spite of this initial improvement, at the end of the study, we did not observe a significant reduction of rejection and it could be because the regenerative potential of MSC was insufficient to revert back the continuous insult of our chronic *in vivo* model. It is important to highlight that *in vivo* models for MSCs are more adverse than *in vitro*, and a poor micro-environment such as low oxygen, inflammatory condition, and free radicals could decrease the nephroprotective effect of MSCs (Chung, 2019). In our renal transplant model, we also applied donor-derived AD- and BM-EVs as an alternative free-cell-therapy. However, in our protocol, the timing and dose of MSC-EVs did not reproduce the results obtained by MSCs. Although the administration of MSC-EVs has achieved success in acute *in vivo* models, many questions about the mechanism of action remain without an answer in chronic models. The design of *in vivo* models could be very complex due to the increased combination of variables in terms of administration timing, cell type or derivatives, the cell number, and administration route, which makes the translation to the clinic difficult.

Our previous results in kidney transplantation using gold standard immunosuppression (IS), calcineurin and mTOR inhibitors, showed a significant reduction of graft damage without reaching complete remission of rejection signs (Rovira et al., 2018). In this study, we aimed to analyze the long-term impact of different cell therapies without the presence of IS on kidney transplantation preventing kidney graft rejection. However, the animal model established and the scheme of cell therapies did not have impressive kidney graft improvement. Further experiments should be performed using immunosuppressive regimens, being mTOR inhibitors our choice, in combination with better cell therapy schemes in order to obtain better results. Inline, Reinders et al. are currently recruiting patients in a Phase II study (NCT02057965) where hypothesizes that the combination of autologous bone marrow MSCs with Everolimus might be an optimal strategy to facilitate early Tacrolimus withdrawal and reduce fibrosis compared to standard Tacrolimus dose (Reinders et al., 2014).

## Conclusion

In view of our results, EVs treatments did not exert any benefit in our experimental settings. In the autologous setting, BM-MSCs prompted as a potentially promising therapy to improve kidney graft outcomes in rats with chronic mixed rejection. On the other hand, in the donor-derived setting, AD-MSC in renal transplantation should be discouraged because accelerated the progression to end-stage kidney disease. Further experiments are required to adjust timing and dose for better long-term outcomes in renal transplantation.

## Data Availability Statement

The datasets generated for this study are available on request to the corresponding author.

## Ethics Statement

The animal study was reviewed and approved by the Comitè Ètic d’Experimentació Animal, CEEA, Decret 214/97, Catalonia, Spain.

## Author Contributions

MR-B, JR, JC, and FD contributed to the conception and design of the study. MR-B, JR, ML-R, EB-M, VT, DM-R, and NH-G contributed in the acquisition and analysis of data for the work. MR-B, JR, PV-A, and FO contributed in the interpretation of data for the work. MR-B and JR wrote the first draft of the manuscript. All authors contributed to the manuscript revision, read, and approved the submitted version.

## Conflict of Interest

The authors declare that the research was conducted in the absence of any commercial or financial relationships that could be construed as a potential conflict of interest.
