# Supplementary material for: Impact of Mesenchymal Stromal Cells and Their Extracellular Vesicles in a Rat Model of Kidney Rejection
Source: Front Cell Dev Biol. 2020 Jan 29;8:10. doi: 10.3389/fcell.2020.00010 (PMC7000363; doi:10.3389/fcell.2020.00010)
Supplement: Supplementary file 1 [file Data_Sheet_1.docx]

Supplementary Material

## Supplementary material: MISEV2018 checklist

Done

**1-Nomenclature**

Mandatory

- Generic term extracellular vesicle (EV): With demonstration of extracellular (no intact cells) and vesicular nature per these characterization (Section 4) and function (Section 5) guidelines OR
- Generic term, e.g., extracellular particle (EP): no intact cells but MISEV guidelines not satisfied

Encouraged (choose one)

- Generic term extracellular vesicle (EV) + specification (size, density, other)
- Specific term for subcellular origin: e.g., ectosome, microparticle, microvesicle (from plasma membrane), exosome (from endosomes), with demonstration of the subcellular origin
- Other specific term: with definition of specific criteria

**2-Collection and pre-processing**

***Tissue Culture Conditioned medium (CCM, Section 2-a)***

General cell characterization (identity, passage, mycoplasma check…). Medium used before and during collection (additives, serum, other)

- exact protocol for depletion of EVs/EPs from additives in collection medium
- Nature and size of culture vessels, and volume of medium during conditioning
- A T150 flask with 15 ml of medium without FBS was used during conditioning
- specific culture conditions (treatment, % O2, coating, polarization…) before and during collection
- Number of cells/ml or /surface area and % of live/dead cells at time of collection (or at time of seeding with estimation at time of collection)
- BM-MSCs
- 5x10^5^ cells/15ml were seeded in a T150 per condition with estimation at time of collection of ± 1,5x10^6^ cells and ±95% of live cells.

- AD-MSCs

- 4.5 x10^5^ cells/15ml were seeded in a T175 per condition with estimation at time of collection of ± 5.3x10^6^ cells and ±96% of live cells.
- Frequency and interval of CCM harvest
- 16 h.

***Storage and recovery (Section 2-d)***

- Storage and recovery (e.g., thawing) of CCM, biofluid, or tissue before EV isolation (storage temperature, vessel, time; method of thawing or other sample preparation)
- The CCM was stored at 4˚C before starting the experiments. After 16 h, the recovered CCM was used at 4°C during centrifugations.
- Storage and recovery of EVs after isolation (temperature, vessel, time, additive(s)…)
- After EVs isolation, samples were resuspended in medium RPMI1640 supplemented with 10% dimethyl sulfoxid, and frozen at -80ºC for the following applications.

**3-EV separation and concentration**

***Experimental details of the method***

- Centrifugation: reference number of tube(s), rotor(s), adjusted k factor(s) of each centrifugation step (= time+ speed+ rotor, volume/density of centrifugation conditions), temperature, brake settings
- Reference number of tubes: Polypropylene Centrifuge Tubes, Beckman Coulter 337986.
- Each tube contained 30ml of CCM.
- Rotor: SW32Ti
- Centrifugation steps:
- 3000 g for 20 min at 4˚C
- Supernatants filtered through 0.22 μm pore filter
- Samples ultracentrifuged (Optima L100XP, Beckman) at 100,000 g for 1 h at 4˚C

**4-EV characterization**

***Quantification (Table 2a, Section 4-a)***

- Volume of fluid, and/or cell number, and/or tissue mass used to isolate EVs NTA
- 30 ml of CCM were used to isolate EVs for NTA
- Global quantification by at least 2 methods: protein amount, particle number, lipid amount, expressed per volume of initial fluid or number of producing cells/mass of tissue
- Ratio of the 2 quantification figures

***Global characterization (Section 4-b, Table 3)Citometria y los marcadores***

- Transmembrane or GPI-anchored protein localized in cells at plasma membrane or endosomes
- The CD63 marker was observed by Flow Cytometry
- Cytosolic protein with membrane-binding or -association capacity
- The CD9 and CD81 markers were observed by Flow Cytometry
- Assessment of presence/absence of expected contaminants
- A total absence of contaminants was observed by Electron Microscopy

 (At least one each of the three categories above)

- Presence of proteins associated with compartments other than plasma membrane or endosomes
- No presence of proteins was observed.
- Presence of soluble secreted proteins and their likely transmembrane ligands
- Topology of the relevant functional components (Section 4-d)

***Single EV characterization (Section 4-c)***

- Images of single EVs **by wide-field and close-up**: e.g. electron microscopy, scanning probe microscopy, super-resolution fluorescence microscopy
- Non-image-based method analyzing large numbers of single EVs: NTA, TRPS, FCS, high-resolution flow cytometry, multi-angle light-scattering, Raman spectroscopy, etc.

**Reporting**

- Submission of methodologic details to EV-TRACK (evtrack.org) with EV-TRACK number provided (strongly encouraged)
- Submission of data (proteomic, sequencing, other) to relevant public, curated databases or open-access repositories
- Data submission to EV-specific databases (e.g., EVpedia, Vesiclepedia, exRNA atlas)
- Temper EV-specific claims when MISEV requirements cannot be entirely satisfied (Section 6-b)

## Supplementary Tables

**Supplementary Table S1:** Flow cytometry antibodies for characterization of MSCs and their EVs.

| **Marker** | **Dye** | **Clone** | **Company** |
| --- | --- | --- | --- |
| CD44H | PE | OX49 | eBioscience |
| CD29 (Integrin β1) | APC | HMb1-1 | eBioscience |
| CD90 | APC | His 51 | Miltenyi Biotec |
| CD45R | PE | His24 | eBioscience |
| CD31 | PE | TLD-3A12 | BD Biosciences |
| MHC-Class I | APC | RT1-A | eBioscience |
| MHC-Class II | PE | His19 | eBioscience |
| CD9 | unstained | H-110 | Santa Cruz |
| CD81 | FITC | Eat2 | Invitrogen |
| Secondary antibody | FITC | Anti-rabbit IgG | Invitrogen |
| Isotype | PE | IgG2a | Thermo Scientific |
| Isotype | APC | IgG | Thermo Scientific |
| Isotype | APC | Arm Ham IgG eBio299Arm | eBioscience |
| Isotype | FITC | IgG1 | Thermo Scientific |

**Supplementary Table S2:** Flow cytometry antibodies for lymphocyte characterization.

| Localization | Marker | Dye | Cat. Nº | Clone | Company |
| --- | --- | --- | --- | --- | --- |
| Surface | CD3 | FITC | 11-0030 | eBioG4.18 | eBioscience |
|  | CD44H | PE | 12-0444 | OX49 | eBioscience |
|  | CD314 (NKG2D) | PE | 12-3140 | 11D5F4 | eBioscience |
|  | CD3 | PerCP-eFluor710 | 46-0030 | eBioG4.18 | eBioscience |
|  | CD161(KLRB1) | PerCP-eFluor710 | 46-1610 | 10/78 | eBioscience |
|  | CD8a | PE-Cy7 | 25-0084 | OX8 | eBioscience |
|  | CD27(TNFRSF7) | PE-Cyanine7 | 25-0271 | LG.7F9 | eBioscience |
|  | CD25 | APC | 17-0390 | OX39 | eBioscience |
|  | CD62L | eFluor660 | 50-0623 | OX85 | eBioscience |
|  | CD45RA | APC-Cy7 | 561624 | OX33 | BD Pharmingen |
|  | IgD | Biotin | MCA190B | MARD-3 | AbD serotec |
|  | Streptavidin | PECy7 | 25-4317 |  | eBioscience |
|  | Streptavidin | APC | 17-4317 |  | eBioscience |
| Intracellular | Foxp3 | PE | 12-5773 | FJK-16s | eBioscience |

## Supplementary Figures

**Supplementary Figure S1.** Representative images of AD- and BM-MSCs characterization by flow cytometry and multi-lineage differentiation potential. **(A)** Flow cytometry analysis of AD- and BM-MSC (blue and red respectively). Both tissue-derived MSC were positive for surface stem cell markers (CD44, CD29 and CD90), and negatives for CD31, and hematopoietic marker (CD45). **(B)** Adipogenic differentiation (x 100 magnification) as demonstrated showing positivity for oil red staining. **(C)** Osteogenic differentiation (x 400 magnification) as demonstrated showing positivity for alkaline phosphatase activity, and was revealed by SigmaFast BCIP/NBT chromogen staining.

**Supplementary Figure S2.** Characterization of AD- and BM-EVs by electron microscopy and Nanoparticle Tracking Analysis (NTA). (**A-B**) Representative cryo-electron microscopy images of AD- and BM-EVs. Images from cryo-electron microscopy (scale bars 0.5 and 0.2um). (**C-D**) NTA measurement shows the concentration and size distribution. The mean size of AD- and BM-EVs was 223.5±84.4nm and 201.0±75.4nm, respectively.

**Supplementary Figure S3.** Characterization of AD- and BM-EVs by flow cytometry. **(A)** Representative microparticle analysis showing Megamix-Plus SSC as internal size standards (160, 200, 240 and 500 nm). **(B, C)** Negative expression of hematopoyetical and endothelial markers, CD45 and CD31 respectively. **(D-F)** Positive expression of mesenchymal markers: CD44, CD90 and CD29. **(G, H)** Positive expression of tetraspanin s as EVs markers: CD9 and CD81.

**Supplementary Figure S4.** Overview of the gating strategy for T, NK and B cells. (**A**) First of all, non-single events (forward scatter area versus forward scatter height) and death cells (LIVE/DEAD Fixable Dead Cell Stain Kits, ThermoFisher) were excluded; gating of lymphocytes (forward scatter area versus sideward scatter area) T, B, and NK cells were detected. (**B**) CD3^+^ cells were used for identification of CD4^+^ T-cells and CD8^+^ T-cells (anti-CD4 versus anti-CD8), whereas CD3^-^ cells were used for identification NK cells (CD314^+^CD161^+^). (**C**) The gated lymphocytes were also used for identification of the B cells. On the left, Dump (-) cells were selected by the exclusion of CD3^+^ and CD161^+^ cells. In the middle, B cells were identified by anti-B220 (CD45RA). On the right, B cells have been differentiated acording IgD staining.

**Supplementary Figure S5.** C4d deposition immunohistochemistry. **(A)** A representative picture of C4d deposition in a kidney graft sample of F-L+Ø group. **(B)** Quantification of C4d deposition in peritubular capillaries. Red arrows indicates peritubular capillaries and black arrows indicates glomerular endothelioum. *Significantly different when compared to F-L+Ø group (*, P<0.05; **, P<0.01).

**

**

**Supplementary Figure S6.** Impact of autologous cell therapies on the amount of cell types into the spleen. **(A)** CD3. **(B)** CD4. **(C)** CD8. **(D)** Natural killer (NK) cells. CD3^-^CDCD314^+^161a^+^ were considered NK cells. (**E**) B cells. CD3^-^CD161^-^B220^+^ were considered B cells.

**

**

**Supplementary Figure S7.** Impact of allogeneic cell therapies on the amount of cell types into the spleen. **(A)** CD3. **(B)** CD4. **(C)** CD8. **(D)** Natural killer (NK) cells. CD3^-^CDCD314^+^161a^+^ were considered NK cells. (**E**) B cells. CD3^-^CD161^-^B220^+^ were considered B cells.
